# Draft Genome Sequence of *Andreprevotia* sp. Strain IGB-42, a Chitinolytic Bacterium Isolated from a Soil Sample of an Anthill in Stuttgart, Germany

**DOI:** 10.1128/MRA.01454-19

**Published:** 2020-03-05

**Authors:** Yevhen Vainshtein, Nicole Werner, Philipp Kirstahler, Karolina Glanz, Christian Grumaz, Thomas Hahn, Susanne Zibek, Kai Sohn

**Affiliations:** a Fraunhofer Institute for Interfacial Engineering and Biotechnology, Stuttgart, Germany; b Institute for Interfacial Process Engineering and Plasma Technology IGVP, University of Stuttgart, Stuttgart, Germany; Broad Institute

## Abstract

*Andreprevotia* sp. strain IGB-42 is a chitin-degrading bacterium isolated from the soil of an anthill. The genome contains 4.7 Mb, a G+C content of 61.31%, 4,257 predicted open reading frames, and a set of industrially interesting chitinase genes.

## ANNOUNCEMENT

Chitin is the second most abundant polysaccharide on Earth after cellulose and is a major structural component of the fungal cell wall and the exoskeleton of arthropods. The main repeating unit is β-1,4-linked *N*-acetylglucosamine, besides a small amount of glucosamine (generally, <10%). Chitin is not soluble in water or many other solvents. This insolubility enormously affects the scalability of the processes for chitin-based products (1, 2). Due to the inertness and crystallinity of chitin, chemical conversion to acid-soluble chitosan requires drastic conditions, rendering the process unsustainable and not ecological (3, 4). The identification of chitinolytic enzymes of microbial origin, enabling chitin conversion under mild conditions, is thus highly sought after.

In a search for enzymes to modify chitin, bacteria were isolated from an enrichment culture from a soil sample of an abandoned anthill located near Lake Baerensee in Stuttgart, Germany. According to Moss et al. (5), the procedure was carried out in sequential cultivation steps, transferring the microorganisms onto plates successively enriching the chitin-utilizing bacteria. This was performed by the gradual substitution of complex medium components and the acid-pretreated colloidal chitin with protein-free and milled chitin as the sole source of carbon and nitrogen. In addition to Amantichitinum ursilacus IGB-41, which was previously described (5, 6), a second Gram-negative bacterial species with chitinolytic abilities was identified. Analysis of the 16S rRNA sequence, isolated and analyzed as described previously (5), revealed that the second isolated species belongs to the genus *Andreprevotia*, with Andreprevotia lacus GFC-1^T^ and Andreprevotia chitinilytica JS11-7^T^ as its closest relatives, with sequence similarities of 98.7 and 97.2%, respectively; it was therefore temporarily named *Andreprevotia* sp. strain IGB-42. A detailed description of *Andreprevotia* sp. IGB-42 is currently in progress and will be published soon.

Here, we describe the draft genome sequence of *Andreprovotia* sp. IGB-42. The library preparation for both the Illumina HiSeq 2000 and Roche GS Junior platforms was done according to standard protocols, as described by Kirstahler et al. (6). All software mentioned was used with default parameters, unless otherwise specified. A total of 3,165,687 reads with a length of 70 bp were generated by Illumina sequencing, and 171,109 reads with a mean length of 449 bp were generated from 454 sequencing, resulting in average genome coverages of 44× and 15×, respectively. Contamination and adapter sequences were removed from the Illumina reads with BBDuk from the BBMap package version 34.41 (http://sourceforge.net/projects/bbmap/). Both Illumina reads and GS reads were used as input for a hybrid assembly with the GS De Novo Assembler (version 2.9), generating 52 contigs with an *N*_50_ value of 311 kb. The average contig length in the presented genome is 78.54 kb. The assembled sequences totaled 4.7 Mb, with a G+C content of 61.31%. Gene annotation was carried out using Prokka version 1.14.1 (7), providing 4,257 potential open reading frames (ORFs), using the following parameters: –kingdom Bacteria –genus “*Andreprevotia*” –species “*Andreprevotia ripae* IGB-42” –strain IGB-42 –locustag IGB-42 –gcode 11 –usegenus –mincontiglen 200 –addgenes –force –compliant –center CDC.

*In silico* analysis to identify the industrially relevant genes for chitin modification resulted in a set of 31 genes, as follows: 27 putative chitinases of the glycoside hydrolase 18 (GH18) family and two of the GH19 family (EC 3.2.14), one putative β-N-acetylhexosaminidase of the GH20 family (EC 3.2.1.52), and one putative chitosanase of the GH46 family (EC 3.2.1.132). Further investigations are now in progress to verify the chitinolytic activity of the newly identified chitinases by heterologous expression and to find promising candidates for industrial applications.

### Data availability.

This whole-genome shotgun project has been deposited at DDBJ/ENA/GenBank under the accession number WXTX00000000. The version described in this paper is version WXTX01000000. The raw LS454 and Illumina data from BioProject accession number PRJNA565654 were submitted to the NCBI Sequence Read Archive (SRA) under the experiment accession numbers SRX6863627 and SRX6863626, respectively.
